# Neutrophilic eccrine hidradenitis secondary to pegfilgrastim in a patient with synovial sarcoma

**DOI:** 10.1002/ccr3.1932

**Published:** 2019-02-07

**Authors:** Neha Puar, Allek Scheele, Francesca Perez Marques, Jyoti Panicker

**Affiliations:** ^1^ University of Kansas Hospital Kansas City Kansas

**Keywords:** febrile neutrophilic dermatosis, granulocyte colony stimulating factor, neutrophilic eccrine hidradenitis, synovial sarcoma

## Abstract

Here, we report a case of neutrophilic eccrine hidradenitis (NEH) in a teenage male with synovial sarcoma associated with extracutaneous manifestations including myositis and splenomegaly secondary to pegfilgrastim. To the best of our knowledge, NEH has not been previously reported to occur in association with extracutaneous manifestations.

## 1 INTRODUCTION

The neutrophilic dermatoses are uncommon in pediatric patients and are characterized by infiltration of neutrophils in the skin and occasionally in extracutaneous tissue.^1^ In neutrophilic eccrine hidradenitis (NEH), the neutrophilic infiltrate has a special predilection for the eccrine glands.^1^ Due to association with fever and similar clinical appearance to cellulitis, the diagnosis of NEH requires a skin biopsy.^2^ Additionally, infectious workup should be performed to rule out infectious etiology because the treatment would be different.^2^ Sweet Syndrome, another type of neutrophilic dermatosis, is classically associated with extracutaneous involvement including the central nervous system, bone marrow, heart, lung, muscles, and spleen.^3^ NEH, however, is not typically associated with extracutaneous disease. Resolution of skin lesions is usually spontaneous; however, recovery occurs much earlier if steroids or NSAIDs are given.

## 2 CASE DESCRIPTION

A 16‐year‐old male with a history of synovial sarcoma of the right posteromedial knee undergoing induction chemotherapy presented with a one‐day history of left upper quadrant abdominal pain and fever. Pain was not associated with eating, stooling, nausea, or vomiting and was only minimally relieved with oxycodone 5 mg. Physical examination revealed left upper quadrant tenderness, however, no guarding or rigidity. There were no other localizing signs of infection. The patient had his port accessed for labs recently, placing him at risk for bacteremia. Initial laboratory workup revealed a white blood cell count of 21 500, C‐reactive protein (CRP) of 3.01, and a normal lactate. Blood cultures and urine culture were also obtained prior to antibiotic initiation. He was hospitalized and started on cefepime. Abdominal tenderness was attributed to constipation due to recent history of hard stools and was treated with a bowel regimen.

**Figure 1:**
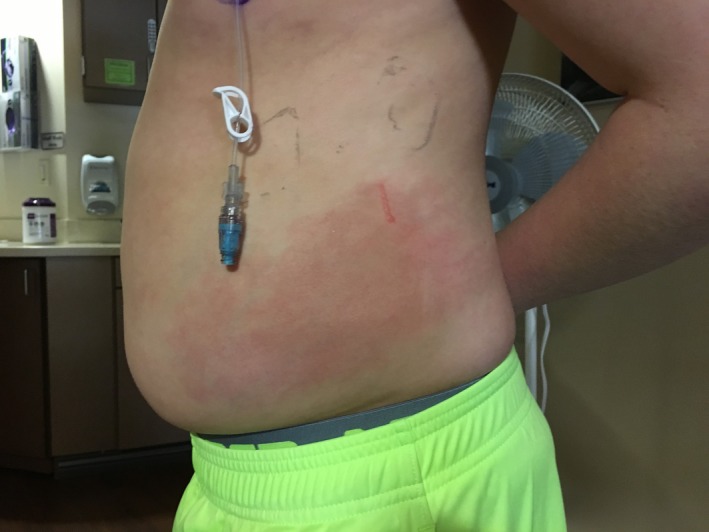
Left sided erythematous abdominal plaque

**Figure 2:**
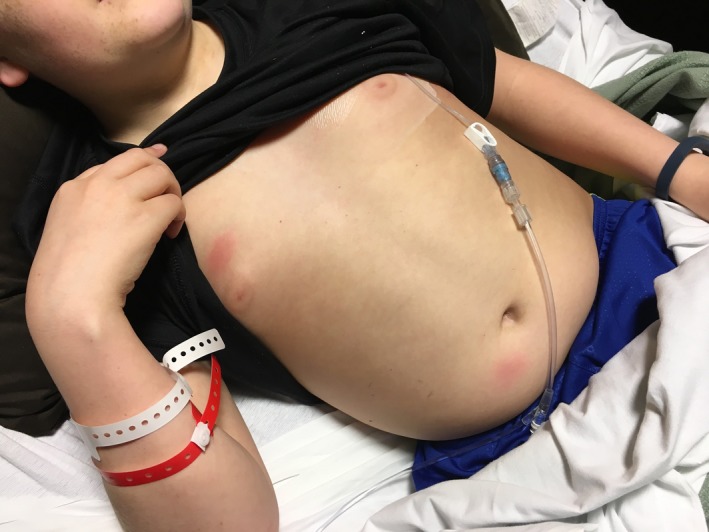
Erythematous plaques on right chest and lower abdomen which developed during previous hospitalization 11 d after G‐CSF administration

**Figure 3:**
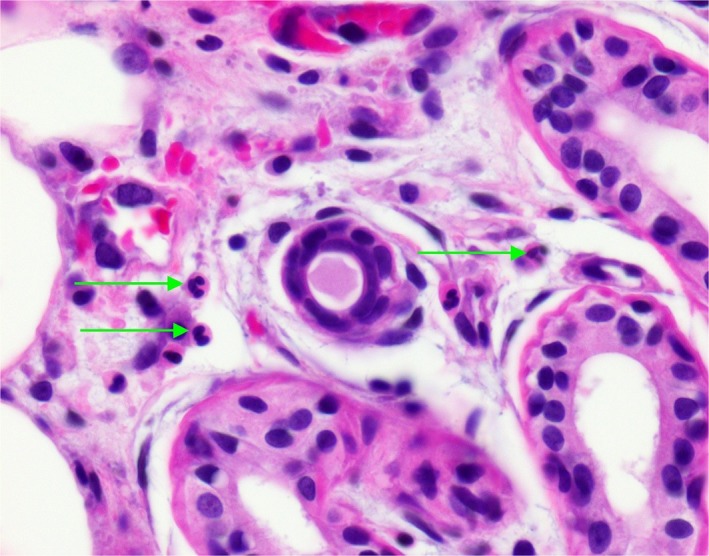
Edema and rare neutrophils in the adventitial dermis around the secretory segment of the eccrine apparatus. Neutrophils noted with green arrows (H/E, X400 original magnification. Skin biopsy from anterior abdominal wall lesion)

After three days of therapy, fever and abdominal pain persisted with a rising white cell count to 38 800 and CRP of 29.86. Blood cultures (including fungal culture) and urine culture showed no growth. Antibiotic coverage was expanded to include vancomycin. Abdominal CT was performed due to concern for an abscess, which revealed moderate retained fecal material, asymmetric thickening and edema of the left lateral abdominal wall musculature, reflecting myositis, and mild splenomegaly, however, no intra‐abdominal abscess. He also developed 2‐3 cm, tender, blanching erythematous patches on his abdomen and upper right arm (Figure 1). Workup was initiated for septic emboli and was negative. New lesions continued to erupt, with expanding size of previous lesions. This included a large plaque on the left abdomen/flank where his previous abdominal pain was located. Further history revealed that a similar lesion occurred on his left chest wall after his second cycle of chemotherapy during an admission for febrile illness and resolved after discharge (Figure 2). Chart review revealed that the patient had received pegfilgrastim twelve days prior to the onset of his current skin lesions and within eleven days of his initial eruption (Table 1). He received doxorubicin and ifosfamide in his first two cycles of chemotherapy and ifosfamide alone during his third cycle of chemotherapy (Table 1). During his hospitalization, he received cefepime for a total of six days and vancomycin for a total of three days. Despite broad‐spectrum antibiotics, he remained intermittently febrile and laboratory workup continued to demonstrate an upward trending CRP. Dermatology was consulted to perform a skin biopsy of his lesions. Per their recommendations, he was started on prednisone therapy to treat presumed acute febrile neutrophilic dermatosis and antibiotics were discontinued. Lesions started to rapidly resolve within 24‐48 hours of therapy initiation. Additionally, CRP started to improve within 48 hours of starting steroids. Dermatopathology revealed sparse neutrophilic infiltrate focally involving the eccrine unit, suggestive of NEH (Figure 3). Culture from the skin biopsy specimen showed no growth of aerobes, fungi, or Mycobacterium tuberculosis. He was discharged home after his clinical condition improved on a two‐week course of oral steroids. Because pegfilgrastim was determined as the likely causative agent, it was discontinued. He subsequently received four more cycles of ifosfamide and doxorubicin without pegfilgrastim and did not have recurrence of his skin lesions (Table 1). In retrospect, his initial eruption was also likely due to NEH given lack of improvement with antibiotics but improvement with steroids which were coincidentally given for nausea.

**Table 1:** Patient's chemotherapy schedule and association with development of NEH

Date	Administered drugs		
	Doxorubicin	Ifosfamide	Pegfilgrastim
1/17/2017	X	X	
1/18/2017	X	X	
1/19/2017		X	
1/21/2017			X
2/6/2017	X	X	
2/7/2017	X	X	
2/8/2017		X	X
2/10/2017			
2/21/2017	a		
2/25/2017		X	
2/26/2017		X	
2/27/2017		X	
3/1/2017			X
3/13/2017	b		
3/24/2017		X	
3/25/2017		X	
3/26/2017		X	
5/8/2017	X	X	
5/9/2017	X	X	
5/10/2017		X	
5/31/2017	X	X	
6/1/2017	X	X	
6/2/2017		X	
6/21/2017	X		
6/22/2017	X		

a Admission for fever with similar, but less intense rash which resolved likely secondary to steroids given for nausea.

b Development of skin lesions seen in NEH described in case report.

## 3 DISCUSSION

Neutrophilic eccrine hidradenitis is a rare neutrophilic dermatosis characterized by the development of tender, edematous, erythematous papules and plaques.^1^ Histologic examination shows neutrophilic infiltration of the eccrine unit and necrosis of the eccrine coils and glands.^2^ It has been associated with malignancies such as acute myelogenous leukemia (AML), Wilms’ tumor, testicular carcinoma, osteosarcoma, Hodgkin's disease, and Non‐Hodgkin's lymphoma.^4^ Occurrence in the setting of synovial sarcoma, to our knowledge, has not previously been described. It has also been associated with chemotherapeutic agents including cytarabine, anthracyclines, bleomycin, cisplatin, and methotrexate.^3^ Seventy percent of chemotherapy‐related NEH occur after the first course of chemotherapy.^3^ Our patient did not develop NEH until after his second cycle of doxorubicin (an anthracycline) and moreover, received anthracyclines later in his course without development of NEH, making this less likely the cause of his NEH. Association with ifosfamide has not been previously described. Infections (HIV, Nocardia, Serratia, and Staphylococcus), Granulocyte colony‐stimulating factor (G‐CSF), systemic lupus erythematosus, and Behcet disease are other associated etiologies.^1^, ^5^, ^6^ In our patient's case, infection was unlikely the cause of his NEH as his blood cultures remained negative during his course and CRP continued to climb despite broad‐spectrum antibiotics, only improving after steroid administration.

### 3.1 Pathophysiology

Neutrophilic eccrine hidradenitis secondary to chemotherapy has been thought to represent a reaction to toxic drugs excreted in sweat.^5^ It has been suggested that G‐CSF induced NEH occurs secondary to enhanced chemotaxis of neutrophils and stimulation of neutrophilic progenitors.^5^ In healthy children with NEH, destruction of immature eccrine glands by high temperatures with resultant inflammatory infiltrate has been proposed as an additional pathogenetic mechanism.^7^ Another hypothesis is that NEH may be a hypersensitivity reaction within the spectrum of other neutrophilic dermatoses such as Sweet syndrome.^8^

### 3.2 Clinical presentation

Patients classically present with tender, erythematous and edematous papules and plaques and rarely, with purpuric lesions.^5^ Sites of involvement include the upper trunk, upper limbs, face (especially the periorbital area), palms, and soles.^1^, ^2^ The axillae and inguinal folds are classically spared. Many patients also present with a fever which may precede the onset of the lesions.^4^ Extracutaneous manifestations including myositis and splenomegaly (as seen in our patient) have been previously reported in Sweet syndrome, however, not in NEH.^3^, ^9^

### 3.3 Diagnosis

Neutrophilic eccrine hidradenitis can mimic cellulitis, and therefore, infection should be ruled out. The diagnosis is histologic, requiring a punch biopsy.^2^ The primary histologic feature is neutrophilic infiltrate in and around the eccrine glands.^1^ In neutropenic patients, the degree of infiltrate may be minimal. Therefore, in such patients, degenerative changes of the eccrine glands may be helpful in making the diagnosis.^1^ If a hematologic malignancy is suspected, a complete blood count should be obtained. In addition, medication intake should be reviewed for potential causative agents.^1^

### 3.4 Treatment

Lesions typically resolve spontaneously within a few days or weeks of discontinuation of causative medications.^5^ Oral corticosteroids, colchicine, anti‐pyretics, and other non‐steroidal anti‐inflammatory drugs have been found to accelerate resolution.^1^, ^3^, ^10^

## 4 CONCLUSIONS

Neutrophilic eccrine hidradenitis is an acute febrile neutrophilic dermatosis associated with malignancies, chemotherapeutic agents, infections, and G‐CSF.^1^ Only a few cases of NEH have been previously reported in the pediatric population. NEH may clinically appear similar to infection and therefore must be distinguished by blood cultures and skin biopsy to guide appropriate therapy.^2^ Extracutaneous involvement, although seen in Sweet Syndrome, has not been previously reported in NEH.^3^, ^9^ Our patient with synovial sarcoma was a unique case of skin biopsy proven NEH who also developed extracutaneous involvement in the form of myositis and splenomegaly, likely secondary to G‐CSF.

## CONFLICT OF INTEREST

The authors listed have indicated they have no potential conflict of interests to disclose.

## AUTHOR CONTRIBUTION

NP and AS: drafted the initial manuscript and reviewed and revised the manuscript. JP and FPM: conceptualized the case report and critically reviewed and revised the manuscript for important intellectual content. All authors approved the final manuscript as submitted and agree to be accountable for all aspects of the work.
